# Effects of Dietary Protein and Starch Digestion Rates on Jejunal and Cecal Microbial Community States and Feed Efficiency in Raccoon Dogs

**DOI:** 10.3390/ani16162549

**Published:** 2026-08-15

**Authors:** Weixiao Nan, Runyu Jiang, Xing Duan, Sibo Chen, Shaochen Yu, Ruijia Deng, Yunxi Zhang, Daozhi Wang, Haoxuan Xu, Zhipeng Li, Huazhe Si

**Affiliations:** 1 College of Animal Science and Technology, Jilin Agricultural University, Changchun 130118, China; nanweixiao@jlau.edu.cn (W.N.); jry030730@126.com (R.J.); dx010726@163.com (X.D.); q1059019684@163.com (S.C.); ysc19940401@163.com (S.Y.); drj020409@163.com (R.D.); zyun_xi@163.com (Y.Z.); wdz2205100709@163.com (D.W.); xuhx1219@gmail.com (H.X.); 2 Joint International Research Laboratory of Modern Agricultural Technology, Ministry of Education, Jilin Agricultural University, Changchun 130118, China

**Keywords:** raccoon dog, community state type, co-occurrence network, feed efficiency, mediation analysis

## Abstract

Feed efficiency remains a significant challenge in farmed raccoon dogs, and performance differences can occur even when diets are formulated with similar nutrient levels. Beyond nutrient concentrations, digestion kinetics can alter the timing of carbon and nitrogen availability along the gastrointestinal tract, thereby influencing microbial assembly in a segment-specific way. Twenty 2-month-old male raccoon dogs (*Nyctereutes procyonoides*) were divided into four dietary groups (n = 5) in a 2 × 2 factorial design. The design combined protein-source formulations containing either soybean cake or dried distillers grains with solubles and starch-source formulations based primarily on either extruded corn or high-amylose corn starch, representing relatively rapid and slow expected digestion characteristics, respectively. The purpose was to assess how digestion-rate synchronization affects jejunal and cecal microbiota structure and whether community state types (CSTs) link diet and feed efficiency. Growth performance varied modestly among treatments, with group B showing the lowest feed-to-gain ratio (F/G) and highest average daily gain (ADG). Starch source significantly affected ADG, while protein and protein × starch interaction influenced F/G. Microbiota profiles were strongly segment-dependent: the cecum had higher richness and diversity than the jejunum. Two-way analyses and Bray–Curtis ordinations showed that the jejunal community was primarily associated with the protein × starch interaction, whereas the cecal community was associated mainly with the protein-source factor and the protein × starch interaction. Network analysis revealed more complex and densely connected microbial communities in the cecum, with core genera occupying central positions. Dirichlet multinomial mixture modeling, performed independently for each intestinal segment, identified two jejunal community states (J-CST1 and J-CST2) and two cecal community states (C-CST1 and C-CST2). J-CST1 and J-CST2 represented alternative lactic acid bacteria-dominated configurations, with J-CST2 enriched in *Lactobacillus* and J-CST1 characterized by higher abundances of *Streptococcus* and *Bacillus*. C-CST2 was represented predominantly by samples from group B and was characterized by higher abundances of *Segatella* and taxa affiliated with *Bacteroidota* and *Lachnospiraceae*. Predicted functional profiles differed between CSTs, involving pathways related to carbohydrate, amino acid, lipid, and bile acid metabolism. Mediation analysis did not provide consistent evidence of significant indirect effects for J-CST membership, whereas C-CST membership was associated with significant estimated indirect effects on final body weight, ADG, and F/G. These findings suggest that dietary protein- and starch-source combinations with different expected digestion characteristics drive segment-specific microbial patterns. Supported by taxonomic, network and functional evidence, cecal CSTs act as an intermediate ecological phenotype that partially explains the variation in growth efficiency, highlighting hindgut community states as potential targets for personalized nutrition of raccoon dogs.

## Simple Summary

Animals may grow differently even when diets contain similar amounts of nutrients. This study examined whether the speed at which protein and starch are digested affects gut bacteria and feed use. Different protein sources have varying molecular structures and processing traits, whereas starch sources differ in amylose content and granule structure; these characteristics influence how quickly digestive enzymes break them down. Four diets were prepared using protein and starch sources that were digested at different rates. We found that the bacterial communities in the small intestine and cecum, which is a fermentation site at the beginning of the large intestine, responded differently to these diets. The small intestine was mainly affected by the combined digestion pattern of protein and starch, while the cecum showed clearer bacterial community patterns related to protein digestion. One diet, combining slower protein digestion with faster starch digestion, was associated with better feed efficiency and a distinct cecal bacterial pattern. These findings suggest that feed formulation should consider not only nutrient levels but also when nutrients become available in the gut. These findings may help guide feeding strategies to optimize feed use and conserve resources in raccoon dog farming.

## 1. Introduction

Farmed raccoon dogs are economically important fur-bearing animals, and feed costs remain a major constraint on profitable and sustainable production [1]. Therefore, improving feed efficiency and growth performance has become an important objective in raccoon dog nutrition. As omnivorous canids, raccoon dogs are adapted to diets containing both animal- and plant-derived ingredients and exhibit a substantial capacity to utilize dietary carbohydrates, although nutrient digestibility may vary with ingredient source and diet composition [2]. Protein and starch are primarily hydrolyzed and absorbed in the small intestine, whereas fractions that escape enzymatic digestion enter the cecum and colon, where they provide substrates for microbial fermentation and may shape hindgut microbial communities [3,4]. Consequently, differences in the digestive characteristics of dietary ingredients may affect not only nutrient absorption but also the quantity and type of substrates available to the hindgut microbiota. This may partly explain why feed conversion and growth performance can differ even when diets are formulated to meet comparable nutrient specifications [5]. Animal performance is regulated not merely by dietary nutrient concentrations, but also by the rate, site, and extent of gastrointestinal nutrient digestion [6]. However, whether different combinations of protein and starch digestion characteristics are associated with nutrient utilization and intestinal microbial ecology in raccoon dogs remains unclear.

Digestion synchrony refers to the extent to which protein and starch digestion are temporally coordinated, thereby influencing the relative availability of amino acids and glucose along the gastrointestinal tract [5,7]. Differences in this coordination may alter the balance of nitrogen- and carbohydrate-derived substrates available to microbial communities in different intestinal segments [8,9]. Gut microbiota are also sensitive to the timing and pattern of nutrient supply, with feeding frequency and intermittent nutrient delivery capable of altering microbial dynamics and host metabolism [10]. Such responses are expected to be segment-specific because the small intestine is characterized by rapid substrate turnover and strong environmental selection, whereas the cecum and colon provide more favorable conditions for fermentation of undigested carbohydrates and nitrogenous substrates [4,11,12,13]. Source-dependent differences in nutrient utilization have also been reported in other farmed carnivores. In blue foxes, protein sources differed in ileal and total-tract crude protein and amino acid digestibility [14], while in mink, protein and amino acid availability varied among fish meal-, poultry meal-, and lamb meal-based diets [15]. These findings support the importance of ingredient source and digestive characteristics, although their effects may differ among species.

The gut microbiota may influence feed efficiency by regulating hindgut fermentation and converting residual dietary substrates into metabolites that interact with host energy and nitrogen metabolism [16]. Carbohydrate fermentation mainly produces short-chain fatty acids, including acetate, propionate, and butyrate, which are absorbed by the host and contribute to energy metabolism [17]. In contrast, microbial fermentation of undigested proteins yields branched-chain fatty acids, ammonia, indoles, and phenolic compounds [18]. Changes in microbial structure may shift the balance between saccharolytic and proteolytic fermentation, affecting energy recovery, the intestinal environment, and nutrient use [19]. These responses are likely to be segment-specific because the small intestine, cecum, and colon differ markedly in substrate availability, transit time, and local ecological conditions. Therefore, identifying distinct microbial community states and their associated functions may provide an integrative approach for evaluating the relationship between digestive patterns and feed efficiency.

Conventional microbiome analyses often focus on diversity metrics or individual differentially abundant taxa, which may be difficult to interpret when multiple taxa respond coordinately or when dietary factors interact [20]. Community state approaches, including enterotypes and community state types (CSTs), provide a complementary means of summarizing complex microbial communities into a limited number of recurrent configurations [21,22]. The Dirichlet multinomial mixture (DMM) model provides a probabilistic framework for identifying such community configurations from sequencing count data. In the present study, we used a 2 × 2 factorial design to investigate the associations between protein- and starch-source combinations with different expected digestion characteristics and jejunal and cecal microbial communities in farmed raccoon dogs. DMM-based CST analysis was applied independently to each intestinal segment to determine whether coordinated microbial community configurations were associated with dietary treatments, predicted microbial functions, growth performance, and feed efficiency. We hypothesized that protein- and starch-source combinations with different expected digestion characteristics would be associated with segment-specific microbial community states and that these states would differ in predicted metabolic functions and show associations with animal performance.

## 2. Materials and Methods

### 2.1. Experimental Design, Animals, and Diets

A 2 × 2 factorial experiment was conducted to evaluate the effects of dietary protein and starch digestion rates on growth performance and intestinal microbiota in raccoon dogs (*Nyctereutes procyonoides*). Two protein digestion rates (rapid and slow) and two starch digestion rates (rapid and slow) were used to establish four dietary treatments: diet A, rapid protein and rapid starch; diet B, slow protein and rapid starch; diet C, rapid protein and slow starch; and diet D, slow protein and slow starch. Soybean cake and extruded corn were designated a priori as the relatively rapid-digestion protein and starch sources, respectively, whereas DDGS and high-amylose corn starch were designated as the relatively slow-digestion sources, based on their ingredient characteristics and previously published evidence [3,7,23]. Accordingly, diets A and D were operationally defined as rate-matched combinations, whereas diets B and C were operationally defined as rate-mismatched combinations. Twenty 2-month-old male raccoon dogs with similar initial body weight were randomly assigned to the four treatments, with 5 animals per treatment.

The initial body weight of the animals was 3.11 ± 0.26 kg. Raccoon dogs were housed individually in cages and had free access to water throughout the experiment. The feeding trial lasted 45 d, including a 5 d adaptation period. The experimental diets were formulated to be isoenergetic and isonitrogenous and to meet the nutrient requirements of growing raccoon dogs. The diets differed mainly in the digestion kinetics of protein and starch. The ingredient composition and nutrient levels of the experimental diets are shown in Table 1. All animal procedures were approved by the Animal Ethics Committee of Jilin Agricultural University (approval number: 20240305001).

### 2.2. Sample Collection

Body weight was recorded at the beginning and end of the feeding trial. The amount of feed offered and refusals were recorded daily to calculate average daily feed intake (ADFI). Average daily gain (ADG) and feed-to-gain ratio (F/G) were calculated using the following equations:

ADG (g/d) = (final body weight − initial body weight)/number of trial days

ADFI (g/d) = total feed intake/number of trial days

F/G = ADFI/ADG

At the end of the feeding trial, animals were humanely euthanized according to institutional guidelines. Animals were not fasted before euthanasia, and sampling was conducted approximately 2 h after the morning feeding. Immediately after euthanasia, the gastrointestinal tract was exposed under sterile conditions. Jejunal digesta were collected from the mid-jejunum, and cecal digesta were collected from the cecum. The digesta collected from each intestinal segment were thoroughly homogenized at the time of sampling. Approximately 5 g of the homogenized digesta was transferred into sterile cryotubes, immediately frozen in liquid nitrogen, and stored at −80 °C until DNA extraction.

### 2.3. Microbial DNA Extraction, 16S rRNA Gene Amplification, and Sequencing

Microbial genomic DNA was extracted from jejunal and cecal digesta using the QIAamp Fast DNA Stool Mini Kit (catalog no. 51604; QIAGEN, Hilden, Germany) according to the manufacturer’s instructions. DNA concentration and purity were determined using a BioTek Epoch 2 microplate spectrophotometer (BioTek Instruments, Winooski, VT, USA). The V3 to V4 region of the bacterial 16S rRNA gene was amplified using the universal primers 341F (5′-CCTACGGGNGGCWGCAG-3′) and 806R (5′-GACTACHVGGGTATCTAATCC-3′). Sequencing libraries were prepared using the Illumina TruSeq DNA PCR-Free Library Prep Kit (Illumina Inc., San Diego, CA, USA), and paired-end sequencing was performed on an Illumina MiSeq platform with a read length of 2 × 300 bp.

### 2.4. Bioinformatics and Statistical Analyses

Raw 16S rRNA gene sequence data were processed using QIIME 2 (v2022.11.1) [24]. Amplicon sequence variants (ASVs) were generated by denoising the sequences with DADA2 [25], and taxonomic assignment was performed against the SILVA database (v138) [26]. ASVs classified as mitochondria, chloroplasts, unassigned sequences, or non-bacterial taxa were removed before downstream analyses. Analyses of α-diversity, β-diversity, differentially abundant genera, and visualization were conducted in R using microeco (v1.8.0) [27]. CSTs were inferred separately for jejunal and cecal samples using DMM modeling implemented in the DirichletMultinomial package (v1.50.0) [21]. Ordination analyses based on Bray–Curtis dissimilarities and the fitting of genus vectors onto ordination space were performed using the vegan package (v2.6-6.1; functions adonis2, metaMDS, cmdscale, and envfit) [28]. Co-occurrence associations were inferred using the SparCC approach implemented via microeco (v1.8.0) and network topological parameters were calculated using igraph (v2.2.1) [29]. Functional profiles were predicted from 16S rRNA gene data using Tax4Fun2 (v1.1.5) [30] to infer KEGG orthologs and pathways. Mediation analyses were conducted using the mediation package (v4.5.1) based on linear models (outcomes) and generalized linear models (mediator), with nonparametric bootstrapping for confidence intervals.

Growth performance, including body weight, ADG, ADFI, and F/G, was analyzed using a two-way analysis of variance in SPSS Statistics, version 22.0 (IBM Corp., Armonk, NY, USA). Before analysis of variance, the normality of model residuals and homogeneity of variance were evaluated using the Shapiro–Wilk and Levene tests, respectively. The statistical model included protein digestion rate, starch digestion rate, and their interaction as fixed effects. When a significant protein × starch interaction was detected, simple comparisons among the dietary combinations were performed using Tukey-adjusted estimated marginal means. When the interaction was not significant, significant main effects were interpreted from the estimated marginal means of the two factor levels. Because each factor had only two levels, no additional post hoc multiple comparison test was required for the main effects. Data were presented as means ± SD. To account for multiple testing, nominal *p* values from the genus-level analyses, predicted KEGG pathway comparisons, and genus-pathway Spearman correlation analyses were adjusted using the Benjamini–Hochberg false discovery rate procedure. For consistency with the figures, the FDR-adjusted values are reported as *p* values throughout these analyses, and an FDR-adjusted *p* value < 0.05 was considered statistically significant.

## 3. Results

### 3.1. Growth Performance of Raccoon Dogs Fed Diets with Different Protein-Starch Digestion Rates

As shown in Table 2, the growth performance of raccoon dogs was affected by dietary protein and starch digestion rates. Initial body weight was comparable among the four treatment groups, ranging from 2.92 to 3.29 kg. After the feeding period, final body weight ranged from 6.09 to 6.54 kg across treatments. Average daily gain was numerically highest in group B at 90.50 ± 11.06 g/d. Starch digestion rate significantly affected ADG (*p* = 0.049), with the estimated marginal mean being higher for the rapid-starch treatments than for the slow-starch treatments (83.00 ± 2.95 vs. 74.13 ± 2.95 g/d). Protein digestion rate and the protein × starch interaction did not significantly affect ADG (*p* = 0.145 and *p* = 0.055, respectively). Both protein and starch digestion rates significantly affected ADFI (*p* = 0.010 and *p* = 0.011, respectively), whereas their interaction was not significant (*p* = 0.110). Estimated marginal means indicated that ADFI was lower with the slow-protein source than with the rapid-protein source (327.35 ± 3.56 vs. 341.93 ± 3.13 g/d) and lower with the slow-starch source than with the rapid-starch source (327.55 ± 3.56 vs. 341.73 ± 3.13 g/d). The feed-to-gain ratio was lowest in group B (3.67 ± 0.43) and highest in group A (4.57 ± 0.23). Protein digestion rate and the protein × starch interaction significantly affected F/G (*p* = 0.017 and *p* = 0.023, respectively, Figure S1), whereas the starch main effect was not significant (*p* = 0.250). Because of the significant interaction, comparisons were focused on the four dietary combinations. Tukey-adjusted pairwise comparisons showed that F/G was significantly lower in group B than in group A (*p* < 0.05), whereas groups C and D did not differ significantly from either group A or group B (*p* > 0.05).

### 3.2. Jejunal and Cecal Bacterial Composition and Diversity

**Figure 1:**
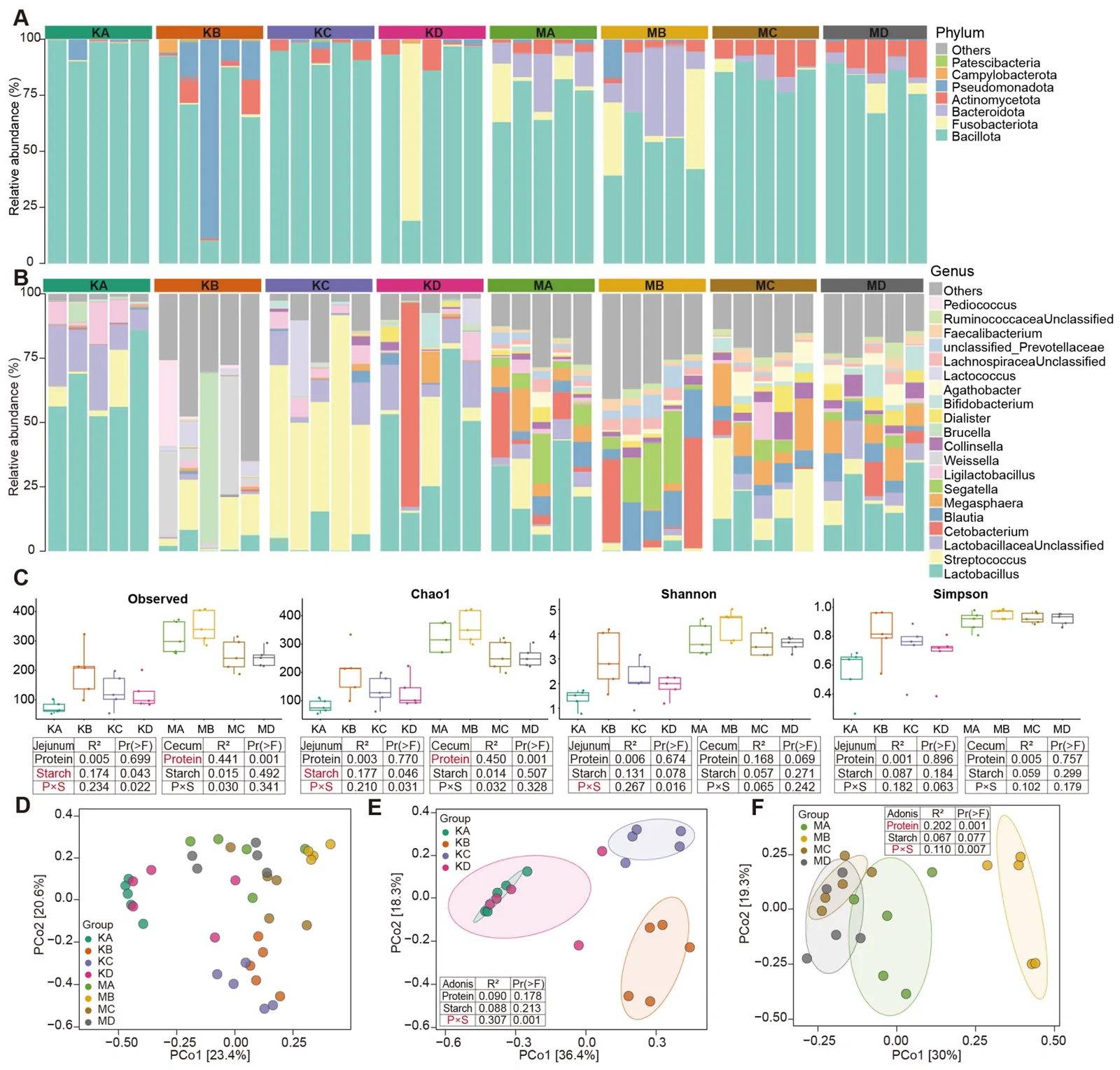
Effects of dietary protein and starch digestion rates on bacterial composition and diversity in the jejunum and cecum of raccoon dogs. KA to KD indicate jejunal samples from groups A to D, and MA to MD indicate cecal samples from groups A to D. Relative abundance of bacterial phyla (**A**) and genera (**B**). For the alpha diversity indices (**C**), the tables below the boxplots show the effects of protein digestion rate, starch digestion rate, and their interaction based on two-way analysis of variance. Principal coordinate analysis based on Bray–Curtis distances for all jejunal and cecal samples (**D**), jejunal samples (**E**), and cecal samples (**F**). Red fonts indicate significant differences at *p* < 0.05.

A total of 2,541,030 valid bacterial sequences were retained from the 40 jejunal and cecal samples, with a mean of 63,526 ± 11,201 sequences per sample and a range of 42,838–88,148 sequences. In the jejunal samples (KA to KD), *Bacillota* was the predominant phylum across all four dietary groups, with mean relative abundances of 97.2%, 65.2%, 94.2%, and 78.3% in KA, KB, KC, and KD, respectively. In KA and KB, *Pseudomonadota* (2.1% and 26.4%, respectively) and *Actinomycetota* (0.4% and 6.1%, respectively) accounted for most of the remaining sequences. In KC, *Actinomycetota* (4.3%) and *Pseudomonadota* (1.0%) were the second- and third-most abundant phyla, respectively. In KD, *Fusobacteriota* had a higher relative abundance than in the other jejunal groups and accounted for 16.0% of the community, whereas *Actinomycetota* (4.4%) remained among the three most abundant phyla. In the cecal samples (MA to MD), *Bacillota* was also the dominant phylum across all treatments, with mean relative abundances of 73.5%, 51.7%, 84.1%, and 80.5% in MA, MB, MC, and MD, respectively. In MA and MB, *Bacteroidota* (13.4% and 24.6%, respectively) and *Fusobacteriota* (8.7% and 16.2%, respectively) were the next most abundant phyla. In contrast, *Actinomycetota* was the second most abundant phylum in MC and MD, with relative abundances of 10.0% and 11.3%, respectively, followed by *Bacteroidota* at 4.3% and 4.1%, respectively (Figure 1A).

At the genus level (Figure 1B) in KA, the most abundant genera were *Lactobacillus* (63.9%), Lactobacillaceae_Unclassified (16.7%), and *Ligilactobacillus* (7.1%). In KB, *Weissella* (17.7%), *Brucella* (13.3%), and *Streptococcus* (11.9%) were the predominant genera. In KC, *Streptococcus* was the dominant genus (58.6%), followed by Lactobacillaceae_Unclassified (8.2%) and *Lactococcus* (6.0%). In KD, *Lactobacillus* was predominant (44.5%), followed by *Cetobacterium* (16.0%) and Lactobacillaceae_Unclassified (10.3%). In MA, the most abundant genera were *Lactobacillus* (24.0%), *Cetobacterium* (8.6%), and *Megasphaera* (8.1%). In MB, the predominant genera were *Cetobacterium* (15.7%), *Segatella* (15.6%), and *Blautia* (13.4%). In MC, *Streptococcus* (16.6%), *Megasphaera* (12.4%), and *Lactobacillus* (10.6%) were predominant. In MD, the most abundant genera were *Lactobacillus* (21.5%), *Megasphaera* (8.2%), and Lactobacillaceae_Unclassified (7.1%).

Alpha diversity analysis showed clear differences in gut microbiota depending on segment and diet (Figure 1C). Cecal samples had higher richness and diversity than jejunal samples, indicating a more complex bacterial community in the hindgut. In both jejunal (KA to KD) and cecal (MA to MD) samples, Group B generally showed the highest richness and diversity. Two-way ANOVA revealed that diet significantly influenced several alpha diversity indices (*p* < 0.05). In the jejunum, starch digestion rate and its interaction with protein digestion affected observed ASVs, Chao1, and the Shannon index. In the cecum, the protein digestion rate significantly influenced observed ASVs and Chao1 (*p* < 0.05).

PCoA based on Bray–Curtis distances further demonstrated a clear separation between jejunal and cecal communities when all samples were analyzed together (Figure 1D). The two segments occupied distinct regions in ordination space, indicating marked segmental differences in microbial community structure. When only jejunal samples were analyzed alone (Figure 1E), the four dietary groups formed partially separated clusters. PERMANOVA further indicated that the interaction between protein and starch digestion rates significantly affected jejunal community composition (R^2^ = 0.30, *p* = 0.001). In the cecum (Figure 1F), community composition was significantly affected by protein digestion rate (R^2^ = 0.20, *p* = 0.001) as well as by the interaction between protein and starch digestion rates (R^2^ = 0.11, *p* = 0.007).

### 3.3. Differential and Core Genera and Co-Occurrence Networks in the Jejunum and Cecum

**Figure 2:**
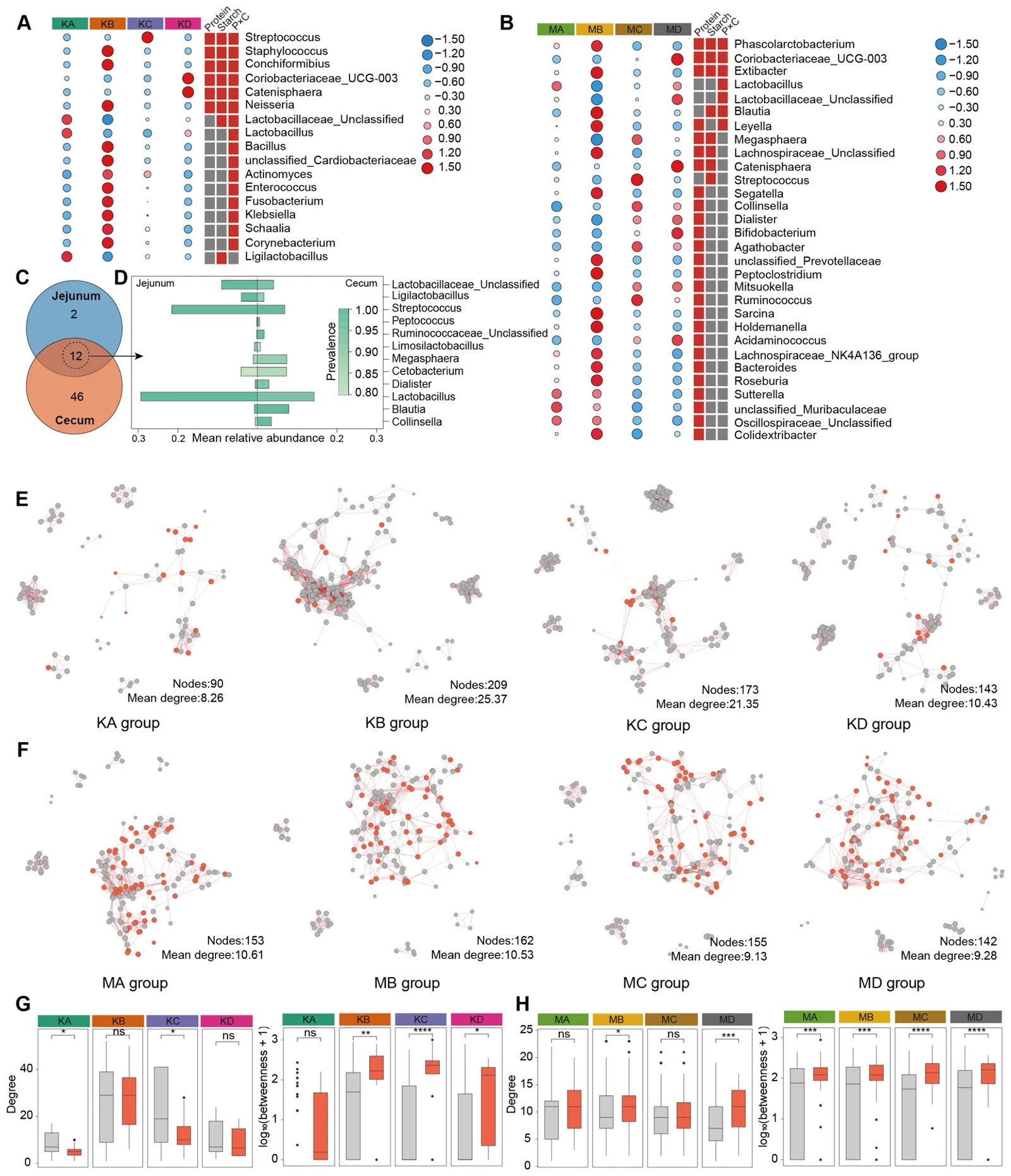
Differential genera, core microbiota, and co-occurrence networks in jejunal and cecal bacteria. KA to KD indicate jejunal samples from groups A to D, and MA to MD indicate cecal samples from groups A to D. Differential genera in the jejunum (**A**) and cecum (**B**) were calculated by two-way analysis of variance. Bubble size and color indicate relative abundance patterns across dietary groups, and the right annotation shows significant effects of protein digestion rate, starch digestion rate, and their interaction. (**C**) Venn diagram showing the numbers of core genera in the jejunum and cecum and the shared core genera between the two intestinal segments. (**D**) Prevalence and mean relative abundance of shared core genera in jejunal and cecal samples. SparCC-based co-occurrence networks of jejunal microbiota in groups KA to KD (**E**) and cecal microbiota in groups MA to MD (**F**); red nodes represent core genera, and other nodes represent non-core genera. Red and blue edges indicate positive and negative associations, respectively. Comparison of degree and betweenness centrality between core and non-core genera in jejunal (**G**) and cecal (**H**) networks. * Asterisks denote significance levels (* *p* < 0.05, ** *p* < 0.01, *** *p* < 0.001, **** *p* < 0.0001).

At the genus level, two-way ANOVA identified 17 jejunal and 30 cecal genera whose abundances were significantly affected by dietary factors, including protein and starch digestion rates and their interaction (*p* < 0.05; Figure 2A,B). In the jejunum, six genera (e.g., *Streptococcus* and *Staphylococcus*) were influenced by protein and starch digestion rates and their interaction (*p* < 0.05). Nine genera (e.g., Lactobacillaceae_Unclassified, *Lactobacillus*, and *Bacillus*) showed significant interaction effects (*p* < 0.05). In the cecum, three genera (e.g., *Phascolarctobacterium* and Coriobacteriaceae_UCG-003) were affected by all three effects (*p* < 0.05). Two genera, including *Lactobacillus*, were affected by the interaction, while 19 (e.g., *Ruminococcus* and Lachnospiraceae_NK4A136) responded only to protein rate (*p* < 0.05). Overall, jejunal genus shifts were mainly due to protein-starch interactions, with 16 of 17 genera showing interaction effects. Most cecal genera responded mainly to protein digestion, with 19 of 30 significant genera. Core-microbiota analysis identified 14 core genera in the jejunum and 58 in the cecum, with 12 shared (Figure 2C; Table S1). Shared genera appeared in most samples, but their mean abundances differed between segments. *Lactobacillus* and *Streptococcus* were more abundant in the jejunum, while *Blautia* and *Megasphaera* were enriched in the cecum (Figure 2D). These findings suggest that the jejunum and cecum share core microbiota but have segment-specific dominant genera, reflecting different microbial niches.

The co-occurrence patterns of jejunal and cecal microbiota were examined using SparCC-based correlation networks. In the jejunum, the four diet-specific networks appeared tight, mainly featuring small to medium clusters of genera with positive correlations and fewer scattered negative links (Figure 2E). Core genera were often central within clusters and connected to multiple non-core taxa. Among these, the KB network showed the most nodes and edges and had the highest average degree, indicating a more complex microbial association pattern. In the cecum, the four networks were larger and more densely connected than in the jejunum (Figure 2F). Core genera tended to occupy central positions. The MB network had the most nodes and a high average degree, forming a dense association cluster. These results indicate that dietary differences in protein and starch digestion rates altered microbiota association structure, with a stronger response in the cecum.

In the jejunal networks, non-core genera generally showed higher degree values than core genera, indicating more direct associations with other taxa, whereas core genera showed significantly higher betweenness centrality than non-core genera in KB, KC, and KD (*p* < 0.05; Figure 2G). These results suggest that non-core genera contributed more to local connectivity in the jejunum, while core genera mainly served as connector taxa linking different parts of the network. In the cecal networks, core genera showed greater topological importance, with higher degrees in most networks and significantly higher betweenness centrality across all four dietary groups (*p* < 0.05; Figure 2H), indicating that core genera played a more central role in maintaining connectivity within hindgut microbial networks.

### 3.4. CST of the Jejunal and Cecal Microbiota, Key Associated Genera and Mediation Effects on Growth Performance

**Figure 3:**
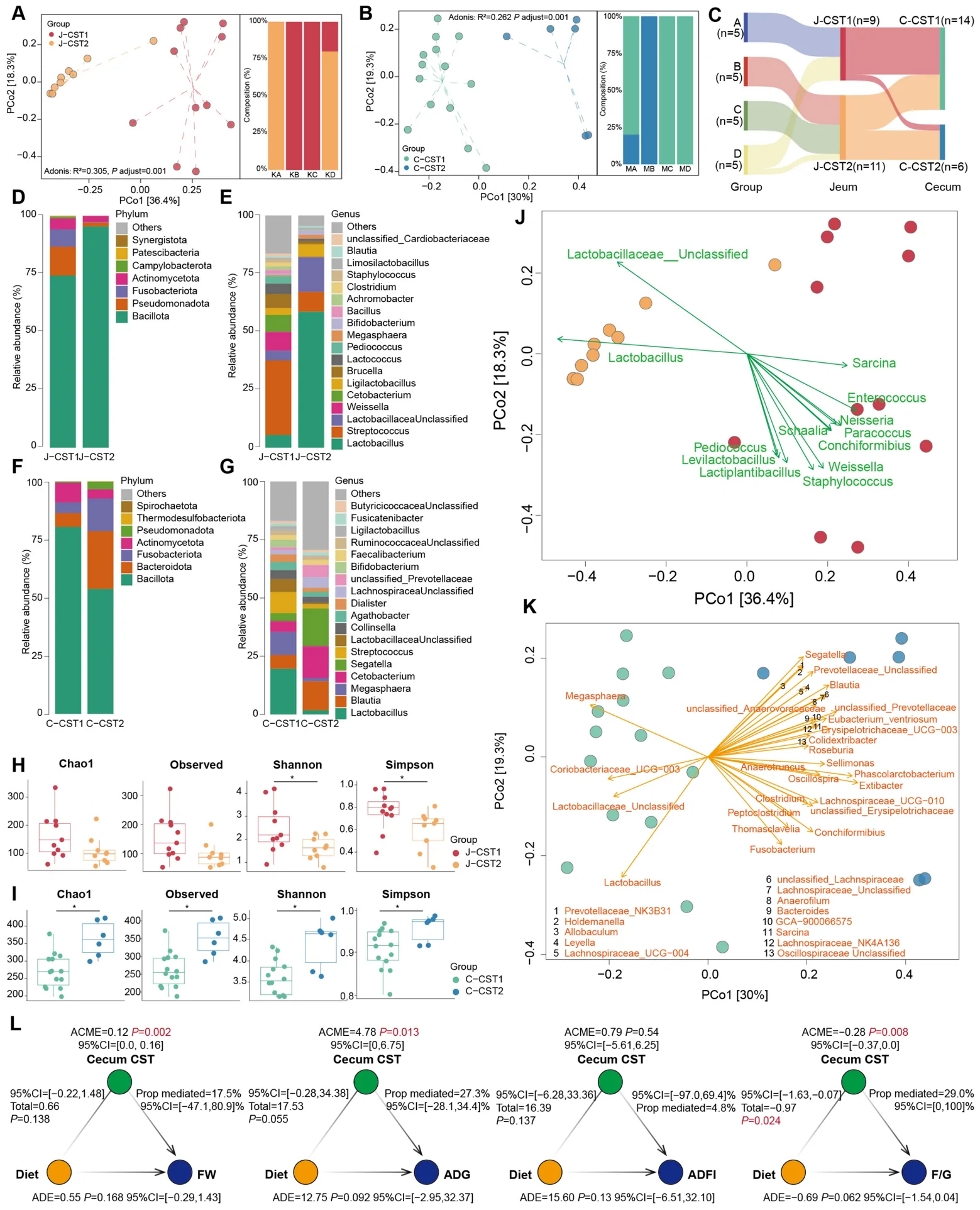
Community state types of jejunal and cecal microbiota and their taxonomic characteristics. Ordination and group distribution of jejunal community state types (**A**) and cecal community state types (**B**). (**C**) Sankey diagram showing the correspondence among dietary groups, jejunal community state types, and cecal community state types within the same animals. Phylum (**D**) and Genus (**E**) level composition of jejunal community state types. Phylum (**F**) and Genus (**G**) level composition of cecal community state types. Alpha diversity differences between jejunal (**H**) and cecal (**I**) community state types. Genus vectors fitted onto the jejunal ordination space (**J**) and cecal ordination space (**K**) using envfit. Only genera significantly associated with the ordination axes were plotted as vectors (*p* < 0.05). Mediation analysis showing the indirect effects of cecal CST membership on growth and feed efficiency traits (**L**). * Asterisks denote significance levels (* *p* < 0.05).

Using DMM modeling, the optimal number of CSTs was identified as 2 for both jejunal and cecal samples based on the Laplace and BIC criteria. In the jejunum, J-CST1 mostly included samples from KB, KC, and one from KD, while J-CST2 included almost all KA samples and the remaining KD samples (Figure 3A). For the cecum, C-CST1 mainly comprised samples from MA, MC, and MD, whereas C-CST2 mainly comprised samples from MB and one sample from MA (Figure 3B). Adonis analysis showed clear separation between CSTs in both segments (R^2^ = 0.305, *p* < 0.001; R^2^ = 0.262, *p* < 0.001). The Sankey diagram showed frequent CST shifts between jejunum and cecum within animals (Figure 3C), indicating that CST assignment depends on the segment and jejunal CST membership did not consistently predict cecal CST membership.

In the jejunum, two CSTs had distinct phylum profiles (Figure 3D). J-CST1 had *Bacillota* at 65.2%, with *Pseudomonadota* (17.2%) and *Actinomycetota* (15.4%). J-CST2 was mostly *Bacillota* (99.7%), with minor *Actinomycetota* (0.2%) and *Fusobacteriota* (0.1%). J-CST2 was marked by lactic acid bacteria, mainly *Lactobacillus* (56.2%), Lactobacillaceae_Unclassified (24.0%) and *Ligilactobacillus* (8.7%). J-CST1 was more diverse, with *Streptococcus* (15.9%), *Bacillus* (8.9%), *Proteocatella* (7.2%), *Clostridium* (6.6%), and *Lactobacillus* (6.2%) as dominant genera (Figure 3E). In the cecum, both CSTs were dominated by *Bacillota*, but their relative abundance profiles differed. In C-CST1, *Bacillota* was the predominant phylum with 77.1%, followed by *Bacteroidota* (15.3%) and *Actinomycetota* (3.6%). In C-CST2, *Bacillota* remained dominant at 64.0%, with *Bacteroidota* increasing to 25.9% and *Actinomycetota* at 6.5% (Figure 3F). At the genus level, C-CST1 was dominated by *Lactobacillus* (21.2%), followed by *Blautia* (9.8%), and *Segatella* (8.7%). In C-CST2, *Segatella* (19.4%), *Blautia* (7.4%) and *Lactobacillus* (6.4%) were the most common (Figure 3G).

Alpha diversity was further compared between CSTs within each intestinal segment (Figure 3H,I). In the jejunum, Shannon and Simpson indices were higher in J-CST1 compared to J-CST2 (*p* < 0.05). In the cecum, observed ASVs, Chao1, Shannon, and Simpson differed significantly between C-CST1 and C-CST2 (*p* < 0.05). These findings show that CST shifts are associated with changes in microbial diversity, especially in the cecum. To identify genera associated with CST separation, an ordination analysis was performed based on Bray–Curtis distances; only genera significantly associated with the axes (*p* < 0.05) were visualized. In the jejunum, samples from J-CST1 and J-CST2 were clearly separated along PCo1 (Figure 3J). *Lactobacillus* and Lactobacillaceae_Unclassified showed long vectors pointing toward J-CST2, suggesting close association (*p* < 0.05). J-CST1 was positioned in the opposite direction along PCo1 and associated with 11 genera, including *Staphylococcus*, *Weissella*, and *Sarcina* (*p* < 0.05). In the cecum (Figure 3K), C-CST1 clustered near Lactobacillaceae_Unclassified, Coriobacteriaceae_UCG-003, *Lactobacillus*, and *Megasphaera* (*p* < 0.05). C-CST2 was associated with a more diverse set of 35 genera, including *Clostridium*, *Bacteroides*, *Fusobacterium*, and several *Lachnospiraceae*, *Prevotellaceae*, and *Oscillospiraceae* (*p* < 0.05).

Mediation analysis with jejunal CST as a mediator yielded inconsistent results for ACME and ADEs (Figure S2), suggesting that jejunal CST likely does not influence growth performance. In contrast, using cecal CST, significant indirect effects were found for final weight (ACME = 0.12, *p* = 0.002), ADG (ACME = 4.78, *p* = 0.013), and F/G (ACME = −0.28, *p* = 0.008) (Figure 3L). The estimated total effect for F/G was −0.97 (*p* = 0.024), suggesting that cecal CST is a potential microbial mediator linking digestion kinetics to feed efficiency. The estimated proportions mediated by cecal CST were 17.5% for final body weight, 27.3% for ADG, 4.8% for ADFI, and 29.0% for F/G. These values represent the estimated proportion of the total effect transmitted through the CST pathway and should not be interpreted as the proportion of variance in each outcome explained by the mediation model.

### 3.5. Predicted Functional Profiles of CSTs and Correlations with Microbial Genera

**Figure 4:**
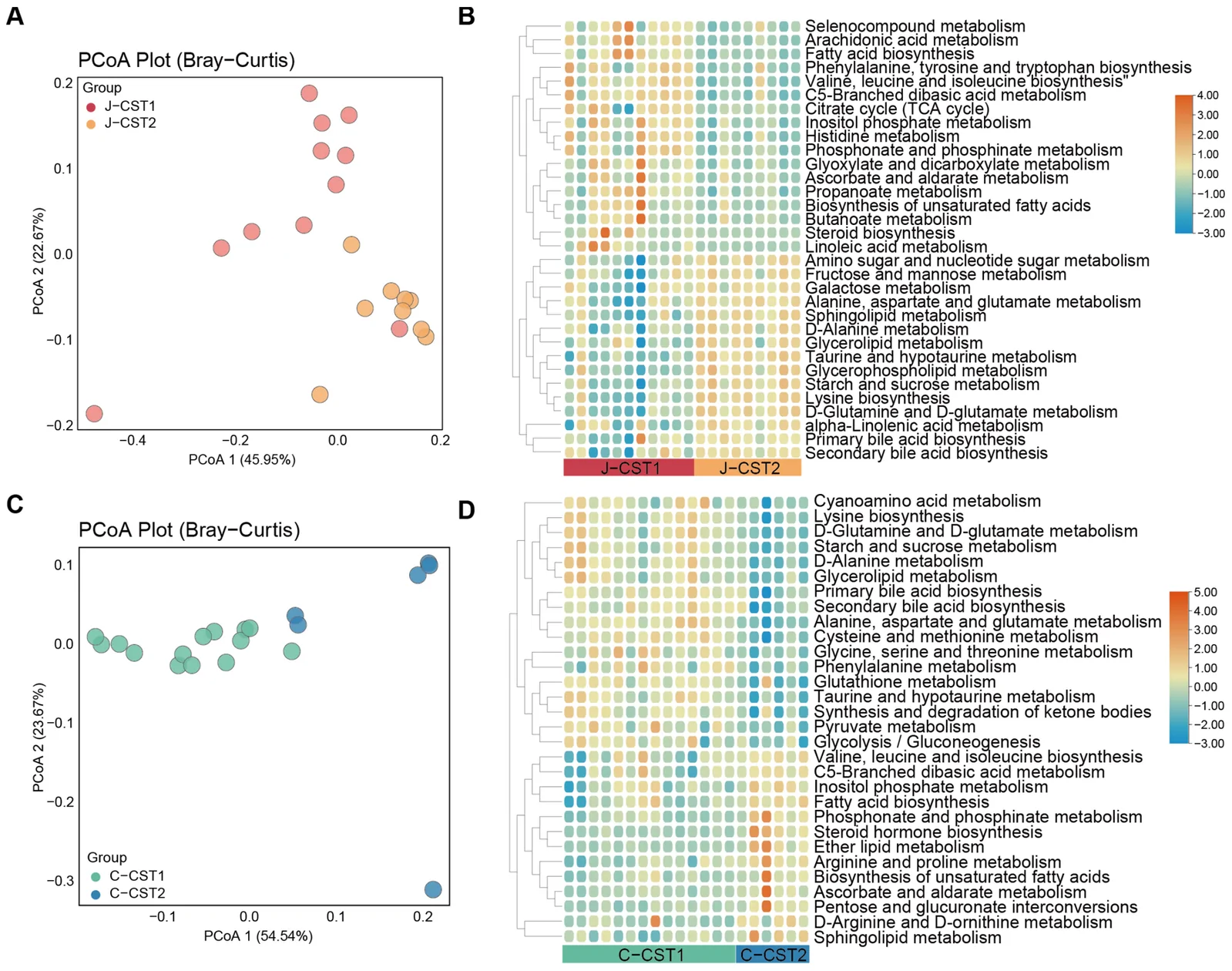
Predicted functional profiles of jejunal and cecal community state types. (**A**) Principal coordinate analysis based on Bray–Curtis distances showing differences in predicted functional profiles between jejunal community state types. (**B**) Heatmap of predicted KEGG pathways differing between jejunal community state types. (**C**) Principal coordinate analysis based on Bray–Curtis distances showing differences in predicted functional profiles between cecal community state types. (**D**) Heatmap of predicted KEGG pathways differing between cecal community state types.

To explore functional differences in CSTs, KEGG pathway profiles were compared between CSTs (Figure 4). PCoA based on Bray–Curtis distances showed clear separation in both the jejunum (Figure 4A) and the cecum (Figure 4C), indicating differences in functional profiles. In the jejunum, the heatmap of predicted KEGG pathways (Figure 4B) revealed that J-CST1 had higher predicted abundances for pathways related to energy and amino acid metabolism, including selenocompound metabolism, arachidonic acid metabolism, and fatty acid biosynthesis (*p* < 0.05). Conversely, J-CST2 showed increased predicted levels of pathways involved in carbohydrate, lipid, and amino acid metabolism, including amino sugar and nucleotide sugar metabolism, fructose and mannose pathways, galactose metabolism, and pathways for alanine, aspartate, glutamate, taurine, starch, lysine biosynthesis, and alpha-linolenic acid biosynthesis (*p* < 0.05). In the cecum (Figure 4D), C-CST1 had higher predicted abundances of pathways related to carbohydrate, amino acid, bile acid, and energy metabolism, including cyanoamino acid metabolism, lysine biosynthesis, D-glutamine and D-glutamate metabolism, bile acid biosynthesis, and pyruvate metabolism (*p* < 0.05). Conversely, C-CST2 showed higher predicted abundances of pathways associated with branched-chain amino acids, lipids, inositol phosphate, steroid hormone, arginine, and sphingolipid metabolism (*p* < 0.05).

**Figure 5:**
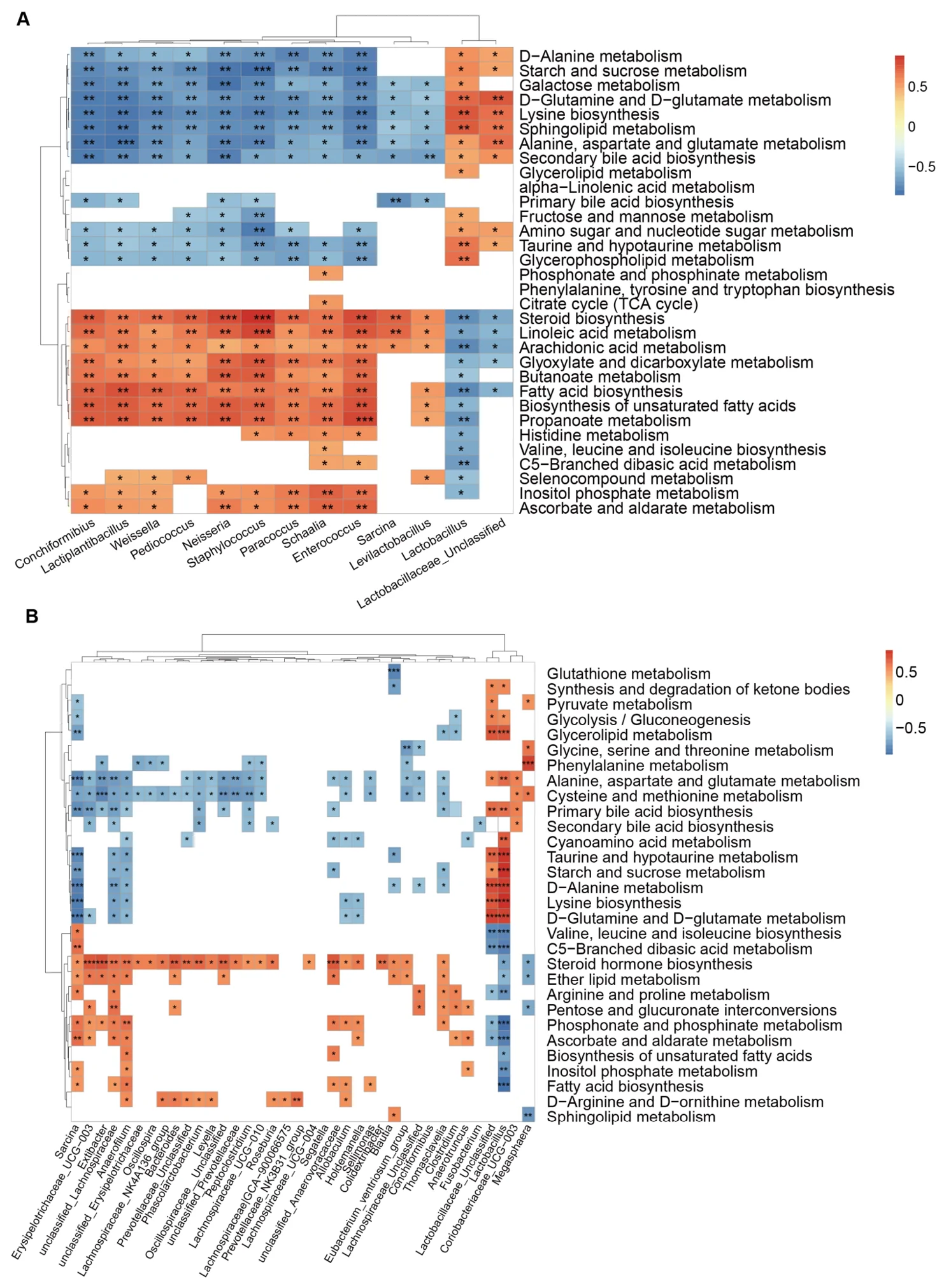
Correlations between key microbial genera and predicted KEGG metabolic pathways in the jejunum and cecum. Spearman correlation heatmaps showing associations between CST-defining genera (identified by envfit analysis) and KEGG pathways in the jejunum (**A**) and cecum (**B**). Only significant correlations are displayed (*p* < 0.05). Positive and negative correlations are indicated by red and blue colors, respectively. * Asterisks denote significance levels (* *p* < 0.05, ** *p* < 0.01, *** *p* < 0.001).

To evaluate associations between CST-defining taxa and predicted microbial functions, Spearman correlation analysis was performed between envfit-identified genera and predicted KEGG pathways. Significant correlations (*p* < 0.05; Figure 5A,B) were visualized. In the jejunum (Figure 5A), *Lactobacillus* and Lactobacillaceae_Unclassified were positively correlated with pathways that were more abundant in J-CST2, including D-glutamine and D-glutamate metabolism, lysine biosynthesis, sphingolipid metabolism, amino acid metabolism, D-alanine metabolism, and starch and sucrose pathways. Conversely, J-CST1-associated genera, including *Staphylococcus*, *Weissella*, and *Sarcina*, correlated positively with CST1-enriched pathways, such as steroid biosynthesis, fatty acid and arachidonic acid metabolism, butanoate metabolism, and propanoate metabolism. In the cecum (Figure 5B), *Lactobacillus* and Lactobacillaceae_Unclassified were positively correlated with taurine and hypotaurine metabolism, starch and sucrose metabolism, and D-alanine metabolism and amino acid biosynthesis. In contrast, Erysipelotrichaceae_UCG-003, *Extibacter*, unclassified_Lachnospiraceae, and *Anaerofilum* were linked to lipid and phosphorus pathways, such as ether lipid metabolism and phosphonate and phosphinate metabolism. These patterns support CST-based functional predictions, with *Lactobacillus*-rich states associated with amino acid, carbohydrate, and bile acid metabolism, while other CST-related genera are linked to fatty acid and lipid pathways.

## 4. Discussion

This study used a 2 × 2 factorial design based on protein and starch sources with different expected digestion characteristics to investigate the relationships among dietary digestion patterns, intestinal microbial ecology, growth performance, and feed efficiency in raccoon dogs. The data showed that the jejunal microbiota was primarily associated with the protein × starch interaction, whereas the cecal microbiota was associated with both the expected protein-digestion-rate category and the protein × starch interaction. Group B showed the numerically highest ADG, the lowest F/G, and a distinct cecal CST. One possible explanation for these segment-specific responses is that differences in digestion characteristics may have altered the temporal and spatial availability of nitrogen- and carbohydrate-derived substrates along the gastrointestinal tract. However, because nutrient disappearance and postprandial substrate availability were not directly measured, this substrate-based mechanism remains hypothetical and requires direct verification.

Growing evidence shows that the gut microbiota responds not only to substrate type but also to the timing of nutrient delivery and feeding schedules. Intermittent nutrient supply can alter microbial rhythms, stability, and metabolic outputs associated with host traits [31,32]. This effect is notable in the small intestine, a quickly responsive segment where lactic acid bacteria fluctuate with diet and protein intake [33]. The study found jejunal bacterial variation mainly related to protein and starch digestion rates, indicating that nutrient timing may be key in this dynamic niche [5,23]. Comparable source-dependent effects have been reported in other carnivorous species. In blue foxes, replacing fish meal with soybean meal, meat meal, or bacterial protein meal altered ileal and total-tract nutrient digestibility [14]. In mink, fish meal showed higher protein and amino acid availability than poultry meal or lamb meal [15]. In dogs, DDGS inclusion and starch source have been reported to affect nutrient digestibility, fermentative metabolites, and fecal microbial composition [34]. Together, these studies support the general premise that ingredient source and physicochemical characteristics can influence nutrient availability and hindgut microbial ecology in carnivorous species. However, the magnitude and direction of these responses may differ among species, and the digestion characteristics assumed in the present raccoon dogs require direct verification.

CST classification and beta-diversity analysis at the taxonomic level identified two jejunal community states enriched in lactic acid bacteria: J-CST1, associated with *Streptococcus* and *Pediococcus*, and J-CST2, associated with *Lactobacillus* and Lactobacillaceae_Unclassified. This pattern is consistent with evidence that nutrient availability and substrate preference can promote the dominance of *Lactobacillus* in the small intestine [35]. Notably, genus-level two-way analysis showed that genera enriched in J-CST2 primarily responded to interactions between protein and starch digestion rates, whereas those in J-CST1 responded primarily to the main effects of these rates. This indicates that different microbial communities respond differently to the timing of nitrogen and carbon availability [36]. This pattern may reflect substrate-specific microbial fermentation. Rapidly available carbohydrates may favor saccharolytic and lactic acid-producing bacteria and promote the production of lactate and short-chain fatty acids [17]. In contrast, relatively greater availability of residual protein under carbohydrate-limited conditions may favor proteolytic fermentation and the production of branched-chain fatty acids, ammonia, indoles, and phenolic compounds [18]. Lactate produced by lactic acid bacteria may also be converted into butyrate through microbial cross-feeding [37]. Because these metabolites were not measured in the present study, these mechanisms should be regarded as possible explanations rather than directly demonstrated processes. The lower alpha diversity observed in J-CST2 was consistent with the marked dominance of *Lactobacillus*, whose small-intestinal predominance may be promoted by substrate-specific nutrient utilization [35]. In dietary networks, core lactic acid bacteria often bridge modules and have higher betweenness centrality than non-core taxa across most diets, suggesting that a few dominant taxa maintain module connections [38]. The KB jejunal network, corresponding to the slow-protein and rapid-starch treatment, showed a highly connected microbial structure.

Compared to the jejunum, the cecum has higher microbial diversity and more active fermentation, likely linked to residual substrates entering the hindgut after digestion [39]. Research shows that starch bypassing small-intestinal digestion influences hindgut fermentation and energy extraction [40], while undigested protein enhances proteolytic fermentation and alters microbial metabolites [41]. Our study found that cecal microbial composition was mainly associated with protein digestion rate and the interaction between protein and starch digestion rates, supporting its role in microbial utilization of undigested carbon and nitrogen sources [42]. Group B exhibited higher alpha diversity than the other groups, possibly due to a more varied residual substrate in the cecum. *Lactobacillus* and Lactobacillaceae_Unclassified, linked to jejunal CST, also dominated cecal CSTs in groups A, C, and D, indicating hindgut environmental filtering may buffer upstream differences in nutrient digestion [43]. The similarity among groups A, C, and D suggests that, despite different proximal digestion, the residual substrates reaching the cecum were similar enough to favor a lactic acid bacteria-based fermentation.

In this intestinal segment, community divergence was mainly associated with the protein-source factor, with 26 of 30 differential taxa responding to protein. This pattern indicates that cecal microbial assembly was more strongly associated with the protein-source factor than with the starch-source factor. One possible explanation is that the protein sources may have differed in the amount or form of nitrogenous substrates reaching the cecum [18]. Despite the convergence of groups A, C, and D, the slow-protein–rapid-starch combination in group B may have altered the relative timing and balance of nitrogen- and carbohydrate-derived substrates reaching the cecum. This pattern may have contributed to the distinct C-CST2 profile, which was characterized by higher relative abundances of *Bacteroidota* and *Segatella* and a lower abundance of *Lactobacillus* than C-CST1. Several protein-responsive genera, including *Acidaminococcus* [44], *Peptoclostridium* [45], *Bacteroides*, and members of *Prevotellaceae* [46], responded significantly to the protein factor. Collectively, these responses suggest that dietary protein source may influence the abundance of taxa involved in nitrogenous-substrate utilization and proteolytic fermentation [45,47].

Compared with the jejunum, the cecum contained more core genera and occupied more central positions in co-occurrence networks, as indicated by higher degree and betweenness centralities. This suggests that cecal community organization may rely more on a core group of fermentative taxa that contribute to network connectivity [48]. This aligns with the idea that core taxa, characterized by high prevalence, play significant roles in maintaining microbiome community structure and stability [49]. Associations between gut microbiome structure and feed efficiency or growth performance have been reported across different animal systems, relating to microbial fermentation, nutrient use, and metabolite production [50,51,52]. Short-chain fatty acids are key mediators linking hindgut fermentation with host energy metabolism [17], while microbially mediated changes in bile acids can influence metabolic signaling and energy balance [53]. In this study, cecal CSTs varied in pathways related to carbohydrate, amino acid, and bile acid metabolism, offering a functional explanation for the association between cecal community states and performance traits.

Several limitations should be considered. First, the relatively small sample size may have limited the statistical power and reduced the precision and stability of the CST classification and mediation estimates. The mediation results should therefore be regarded as exploratory associations rather than evidence of causality. Second, the rapid- and slow-digestion categories were assigned based on ingredient characteristics and published evidence, whereas protein and starch digestion kinetics, ileal digestibility, and postprandial nutrient appearance were not directly measured in the present study. Therefore, differences in nutrient flow to the jejunum and cecum remain inferred rather than experimentally demonstrated. Third, predicted KEGG functions derived from 16S rRNA gene data do not constitute direct measurements of microbial functional genes or metabolic activity. Neither targeted nor untargeted metabolomic validation was performed, and short-chain fatty acids, branched-chain fatty acids, ammonia, bile acids, and other microbial metabolites were not measured. Consequently, the proposed fermentation mechanisms could not be directly validated. Fourth, the microbiota was assessed only at the end of the trial, and the temporal stability of the identified CSTs could not be evaluated. Finally, all animals were young males at a single growth stage; therefore, the findings may not be directly generalized to females, mature animals, breeding animals, or other production conditions. Larger studies incorporating direct measurements of nutrient digestion kinetics and digestibility, metabolomics, fermentation metabolites, and longitudinal microbiota sampling are required.

## 5. Conclusions

This study showed that diets formulated with protein and starch sources differing in their expected digestion characteristics were associated with segment-specific variation in the jejunal and cecal microbiota of young male raccoon dogs. Jejunal community composition was primarily associated with the protein × starch interaction, whereas cecal composition was more closely associated with the protein-source factor and its interaction with starch. Group B exhibited the lowest F/G and a distinct cecal CST. Cecal CST membership was also statistically associated with indirect effects on final body weight, ADG, and F/G, suggesting that cecal community states may represent a potential intermediate ecological phenotype linking dietary treatments with variation in animal performance. These findings suggest that ingredient digestion characteristics, in addition to total nutrient concentrations, may be relevant when formulating diets for raccoon dogs. However, larger studies incorporating direct measurements of nutrient digestion kinetics, digestibility, and microbial metabolites are required to confirm these associations and their underlying mechanisms.

## Figures and Tables

**Table 1:** Ingredients and nutrient levels of experimental diets.

Ingredients	Content %			
	A	B	C	D
Extruded corn	36	36	15	15
High amylose corn starch	-	-	17	17
Soybean cake	13.72	-	14	-
DDGS	-	10	-	13
Rice bran meal	9	9	9	9
Fish meal	9	9	9	9
Soybean meal	13	13	13	13
Corn germ meal	5	5	5	5
Soybean oil	10	10	10	10
NaCl	0.3	0.3	0.3	0.3
DL-Methionine	0.041	-	0.3	0.077
L-Lysine	-	0.926	0.189	1.169
L-Arginine	-	1.01	0.35	0.953
L-Histidine	-	0.329	0.268	0.331
L-Isoleucine	-	0.571	0.293	0.473
L-Leucine	0.439	-	0.9	0.305
L-Cysteine	-	0.074	0.298	0.189
L-Phenylalanine	-	0.436	0.486	0.305
L-Tyrosine	-	0.223	0.342	0.291
L-Valine	-	0.1	0.415	0.212
L-Tryptophan	-	0.324	0.062	0.476
L-Threonine	-	0.207	0.297	0.419
CaHPO_4_	2	2	2	2
Premix ^(1)^	1.5	1.5	1.5	1.5
Total	100.0	100	100	100
Nutrient levels	Content			
ME ^(2)^ (MJ/kg)	15.94	15.95	15.84	15.88
ADF, %	5.86	4.99	5.34	4.79
NDF, %	11.61	12.75	11.70	12.13
Crude fiber, %	3.05	3.03	3.12	2.98
Starch, %	31.88	30.97	31.16	31.38
CP, %	19.90	19.18	19.74	19.10
EE, %	13.21	13.38	13.22	13.10
Ca, %	1.03	1.07	1.04	1.09
P, %	0.95	1.00	0.97	1.01

^(1)^ Per kilogram of compound feed contained the following: VA 60,000 IU, VD_3_ 24,000 IU, VE 100 mg, VK_3_ 10 mg, VB_1_ 12 mg, VB_2_ 50 mg, VB_6_ 20 mg, VB_12_ 0.15 mg, Nicotinamide 200 mg, Folic acid 10 mg, Biotin 0.2 mg, Calcium pantothenate 90 mg, VC 4 mg, Cu 80 mg, Fe 0.16 g, Zn 76 mg, Mn 76 mg, I 1 mg, Se 0.6 mg, Co 0.2 mg. ^(2)^ ME was calculated, whereas the other nutrient values were measured.

**Table 2:** Effects of dietary protein and starch digestion rates on growth performance in raccoon dogs.

Item	Group				*p* Value		
	A	B	C	D	Protein	Starch	P × S
Initial body weight, kg	3.07 ± 0.14	2.92 ± 0.18	3.29 ± 0.30	3.17 ± 0.30	0.235	0.051	0.892
Final body weight, kg	6.09 ± 0.09	6.54 ± 0.53	6.30 ± 0.34	6.09 ± 0.70	0.581	0.581	0.141
Average daily gain, g/d	75.50 ± 4.38	90.50 ± 11.06	75.25 ± 6.86	73.00 ± 12.58	0.145	0.049	0.055
Average daily feed intake, g/d	344.93 ± 8.99	338.52 ± 2.73	338.92 ± 13.42	316.18 ± 8.82	0.010	0.011	0.110
F/G	4.57 ± 0.23 ^b^	3.67 ± 0.43 ^a^	4.38 ± 0.24 ^ab^	4.42 ± 0.52 ^ab^	0.017	0.250	0.023

A, rapid protein and rapid starch; B, slow protein and rapid starch; C, rapid protein and slow starch; D, slow protein and slow starch. P, main effect of protein digestion rate; S, main effect of starch digestion rate; P × S, interaction between protein and starch digestion rates. Values are presented as mean ± SD (n = 5 per treatment). When the protein × starch interaction was significant, different lowercase superscript letters within a row indicate significant differences among dietary combinations based on Tukey-adjusted comparisons of estimated marginal means (*p* < 0.05). Superscript letters are not shown when the interaction was not significant.

## Data Availability

The datasets generated in this study are publicly available in the NCBI repository under the accession number PRJNA1499300.

## Supplementary Materials

The following supporting information can be downloaded at: https://www.mdpi.com/article/10.3390/ani16162549/s1, Table S1: List of core bacterial genera in the jejunal and cecal digesta of raccoon dogs. Figure S1: Interaction between the expected protein and starch digestion-rate classifications for the feed-to-gain ratio of raccoon dogs. Figure S2: Mediation analysis showing the indirect effects of jejunal CST membership on growth and feed efficiency traits.
