# Supplementary material for: Effects of Dietary Protein and Starch Digestion Rates on Jejunal and Cecal Microbial Community States and Feed Efficiency in Raccoon Dogs
Source: Animals (Basel). 2026 Aug 15;16(16):2549. doi: 10.3390/ani16162549 (PMC13508941; doi:10.3390/ani16162549)
Supplement: Supplementary file 1 [file animals-16-02549-s001.zip › Supplementary figures.pdf]

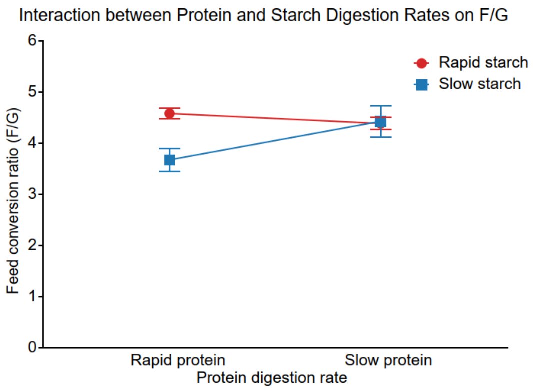

Fig. S1 Interaction between the expected protein and starch digestion-rate classifications for the feed-to-gain ratio of raccoon dogs. Values are presented as mean ± SD.

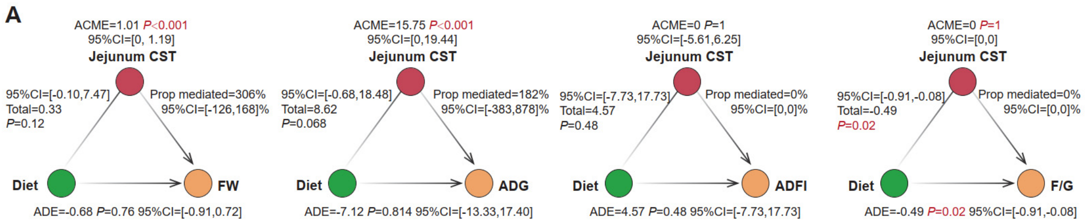

Fig. S2 Mediation analysis showing the indirect effects of jejunal CST membership on growth and feed efficiency trait
